# Plasmonic Optical Tweezers and Surface-Enhanced Raman Spectroscopy: Fundamentals, Single-Entity Applications, and the Evolving Role of Artificial Intelligence

**DOI:** 10.3390/bioengineering13070819

**Published:** 2026-07-16

**Authors:** Xuanzhi Wang, Yuli Lu, Yizhou Zou, Fan Gao, Domna G. Kotsifaki

**Affiliations:** Photonics Lab, Division of Natural and Applied Sciences, Duke Kunshan University, 8 Duke Ave, Kunshan 215316, China; xuanzhi.wang@dukekunshan.edu.cn (X.W.); yuli.lu@dukekunshan.edu.cn (Y.L.); yizhou.zou@dukekunshan.edu.cn (Y.Z.); fan.gao@dukekunshan.edu.cn (F.G.)

**Keywords:** plasmonic optical tweezers, surface-enhanced Raman spectroscopy (SERS), single-molecule detection, single-entity analysis, artificial intelligence (AI), machine learning (ML), nanophotonics

## Abstract

The ability to manipulate and probe individual nano-particles, viruses, and organelles with high sensitivity and specificity is an essential part of modern nanoscience and molecular biology. Plasmonic optical tweezers (POT), which use localized surface plasmons to create nanoscale-confined optical fields, have emerged as a powerful platform for trapping and manipulating single nano–bio entities at low optical powers. When combined with surface-enhanced Raman spectroscopy (SERS) from the same plasmonic nanostructures, these platforms offer a unique multi-modal capability: simultaneous optical manipulation and label-free chemical fingerprinting of a single specimen. However, the field faces critical challenges, including low throughput, thermal noise, photothermal damage, and the overwhelming complexity of interpreting dynamic, single-molecule SERS data. This review examines the transformation of plasmonic optical trapping and spectroscopy as they evolve toward autonomous operation and intelligent decision-making. We begin with the fundamental principles that enable these tools to manipulate and probe single viruses, organelles, and nano-particles. Building on this foundation, we explore how computational intelligence is being integrated into the field to address long-standing challenges. This includes the emergence of data-driven methods for designing optimized plasmonic nanostructures, for decoding the complex molecular fingerprints hidden in single-molecule SERS spectra, and for creating feedback-driven systems capable of adaptive, real-time experiment control. By synthesizing these developments, we illustrate a clear trajectory: from manually operated instruments toward fully integrated intelligent nanophotonic laboratories that can autonomously discover and characterize the nano-world. We conclude by discussing the remaining challenges—from data acquisition and model interpretability to the mitigation of photothermal effects—and the most promising pathways toward realizing this transformative vision for virology, cell biology, and nanomedicine.

## 1. The Era of Single-Entity Nanophotonics

The ability to manipulate and characterize single nanoscale objects [1] has become a major goal across nanoscience, molecular biology [2], and nanomedicine [3,4,5]. In many optical and spectroscopic experiments, the measured signal comes from large populations of particles or molecules, so the outcome represents an average over the entire ensemble. Such averaged signals hide the distinct behavior that individual particles may exhibit. In biological systems and nanomaterials, variations at the single-particle level often lead to differences in properties and responses that influence overall function. Consequently, a major shift has occurred toward single-entity nanophotonics, where light is employed not only as a probe but also as a tool to directly interact with individual nano-particles, viruses, vesicles, and organelles in real-time.

Optical tweezers established a practical method for the non-contact manipulation of microscopic dielectric objects through optical gradient forces [6,7]. Since their first development, they have transformed fields ranging from soft matter physics to molecular biophysics [8]. However, as the characteristic size of the target decreases well below the wavelength of light, the efficiency of conventional optical trapping decreases due to the diffraction limit and the unfavorable scaling of optical forces with particle volume. This limitation affects the applicability of traditional optical tweezers for studying proteins, extracellular vesicles, and synthetic nano-particles in the deep nanoscale regime.

Plasmonic nanostructures provide a way to overcome this barrier [9,10,11]. By exploiting localized surface plasmon resonances (LSPR) supported by metallic nanostructures, optical fields can be confined to nanoscale volumes far below the diffraction limit, generating large electromagnetic field gradients. These gradients are capable of producing sufficient optical forces to trap sub-100 nm objects at low optical powers, thereby establishing the foundation of POT [12,13,14,15,16]. Importantly, plasmonic nanostructures do more than enhance optical forces. The same electromagnetic hotspots responsible for trapping [17] also can amplify optical signals such as Raman scattering [18,19,20], fluorescence [21], and elastic scattering. As a result, the trapping site inherently becomes a sensing site. This dual functionality enables simultaneous manipulation and label-free interrogation of a single nano–bio entity within the same nanoscale region [22]. While similar trapping–spectroscopy combinations have been demonstrated with conventional optical tweezers, POTs provide this capability at much smaller length scales and with significantly enhanced optical sensitivity [1,23].

Moving from ensemble-averaged measurements to single-entity experiments introduces significant data complexity. Each trapping event generates time-resolved information, including positional fluctuations, spectral evolution, and interaction dynamics influenced by thermal and environmental effects [24,25]. Unlike averaged measurements, which produce averaged signals, single-entity experiments yield large datasets that are inherently heterogeneous, time-dependent, and often noisy. The difficulty, therefore, lies in translating these complex, time-dependent datasets into meaningful physical and mechanistic understanding. This growing analytical challenge has motivated the integration of computational approaches, including ML and automated data analysis, into nanophotonic experiments [26,27]. These methods can assist in identifying patterns in noisy data, classifying spectral responses, and enabling data-driven experimental interpretation.

**Figure 1:**
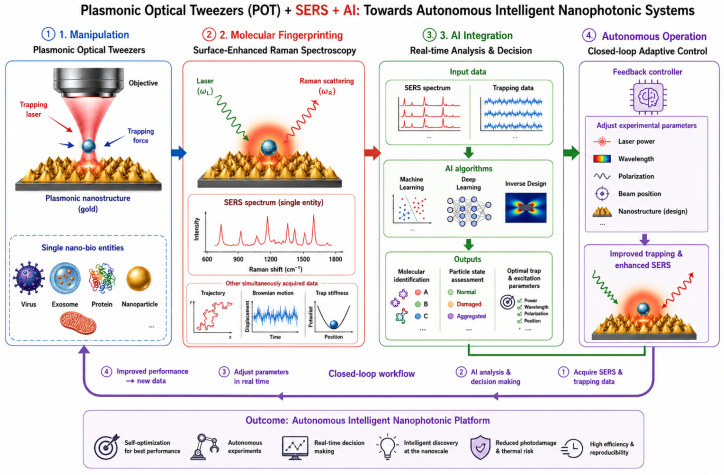
Conceptual roadmap of this review. Plasmonic optical tweezers (POT) enable the manipulation of individual nano–bio entities, while surface-enhanced Raman spectroscopy (SERS) provides simultaneous label-free molecular fingerprinting using the same plasmonic hotspot. The resulting multi-dimensional experimental data are analyzed using artificial intelligence (AI), machine learning (ML), deep learning (DL), and inverse design approaches, enabling automated spectral interpretation, nanostructure optimization, and feedback-controlled experiments. Together, these developments illustrate the evolution of POT-SERS platforms toward autonomous intelligent nanophotonic systems. Figure created with assistance from ChatGPT (OpenAI-GPT 5.5).

As a result, plasmonic optical tweezers and surface-enhanced spectroscopy techniques are evolving into intelligent, adaptive nanophotonic platforms. The role of light at the nanoscale is therefore shifting from passive illumination toward active manipulation and analysis of single nano–bio entities. The present review examines the principles and applications of POTs as tools for confining light at the nanoscale to manipulate and probe single nano-objects. We begin by outlining the physical foundations that govern plasmonic optical trapping and surface-enhanced Raman spectroscopy. We then review how these tools are applied to study synthetic nano-particles, viruses, and organelles. Building on this foundation, we discuss the role of artificial intelligence (AI) in addressing the data complexity inherent in single-molecule experiments and enabling real-time feedback control. Finally, we consider the current challenges and future perspectives toward fully integrated intelligent plasmonic platforms. In this context, unlike earlier studies that primarily addressed optical trapping or Raman spectroscopy independently [1,28,29,30], recent works have begun to explore their integration within hybrid POT-SERS platforms, including plasmonic nanogap-based approaches that enhance both trapping efficiency and Raman signal generation [31]. Therefore, the present review provides a broader perspective by additionally discussing emerging directions toward AI-assisted, adaptive, and autonomous nanophotonic systems [25,27,32], which are not comprehensively covered in earlier integration-focused studies (Figure 1).

## 2. Fundamentals of Plasmonic Optical Tweezers and Surface-Enhanced Raman Spectroscopy (SERS)

The principles of POTs [1,14,29,33,34] and plasmon-enhanced spectroscopy [11,35,36,37] have been extensively discussed in numerous review articles in the literature. These works offer a comprehensive description of the underlying physical mechanisms and their applications across nanophotonics and biophysics. Here, we summarize the key concepts relevant to POT and SERS.

### 2.1. Principles of Plasmonic Optical Tweezers (POTs): Confining Light at the Nanoscale

Conventional optical tweezers generate trapping forces by tightly focusing a laser beam, where the optical gradient force acting on a small dielectric particle in the Rayleigh regime can be approximated as [38,39,40]

(1) $$F_{\mathrm{grad}}=\frac{1}{2}\;\alpha \nabla {\left|E\right|}^{2},$$

where α is the particle polarizability and *E* is the electric-field amplitude. For a spherical particle of radius *r*, the polarizability is given by [38]

(2) $$\alpha =4\pi \varepsilon_{0}r^{3}\left(\frac{m^{2}-1}{m^{2}+2}\right),$$

with $m=n_{p}/n_{m}$ representing the refractive index ratio between the particle and the surrounding medium. These expressions show that the trapping force scales with $r^{3}$, which rapidly reduces the effectiveness of conventional optical tweezers as the particle size decreases to the nanoscale. Confining objects below a few tens of nanometers therefore requires increasing the trapping laser intensity, often to levels that can induce unwanted heating, particularly for biological samples.

This limitation is motivated the exploration of alternative approaches to produce strong optical forces at small length scales. POTs address this challenge by employing metallic nanostructures that support localized surface plasmon resonances (LSPR). Under resonant illumination, the electromagnetic field becomes confined to subwavelength regions near the metal surface, creating localized hotspots characterized by extremely large field gradients. In these regions, the term ${\nabla |E|}^{2}$ can be orders of magnitude larger than in diffraction-limited focusing, significantly enhancing the optical force without requiring high incident trapping power. In this regime, trapping is governed by near-field confinement rather than far-field focusing. The optical potential experienced by a nano-particle can be expressed as [12]

(3) $$U\left(r\right)=-\frac{1}{2}\alpha {\left|E\left(r\right)\right|}^{2},$$

indicating that nanoscale variations of the electromagnetic field translate into deep trapping potentials over nanometric distances. Because the spatial extent of these enhanced fields is comparable to the size of nano-particles and bio-molecules, plasmonic hotspots act as effective nanoscale traps at relatively low optical powers. Note that although POTs are predominantly operated using continuous-wave lasers, nonlinear optical effects may become relevant under high peak-power or ultrafast laser excitation [41,42]. Strong plasmonic field confinement can substantially enhance nonlinear light–matter interactions, including multi-photon absorption, nonlinear polarization, and harmonic generation [42]. While these effects are not typically exploited for conventional optical trapping, they have attracted increasing interest in combining optical manipulation with nonlinear imaging and spectroscopy and may provide additional functionality in future multifunctional trapping platforms.

Nanostructures such as nanoholes [4,13,14,17,43,44,45,46], nano-antennas [15,47], and nano-particle-based assemblies [1], for example, serve as platforms where light is both highly concentrated and spatially structured in a manner ideally suited for manipulating very small objects. It is this near-field mechanism—driven by plasmon-induced field confinement and steep intensity gradients—that enables plasmonic nanostructures to function as optical traps for nano-particles, viruses, and bio-molecules well below the diffraction limit.

### 2.2. Surface-Enhanced Raman Scattering (SERS): Mechanism and Single-Molecule Sensitivity

SERS is a vibrational spectroscopy technique in which the normally weak Raman signal from a molecule becomes strongly amplified when the molecule is located in close proximity to a plasmonic metal nanostructure [11,30,48]. In Raman scattering, the intensity of the scattered light depends on the electric field experienced by the molecule. When a molecule is positioned near a plasmonic nanostructure, the incident field is no longer the original external field but a locally enhanced near-field created by the plasmonic response of the metal. This local field can be written as [48]

(4) $$E_{\mathrm{loc}}=M\left(\omega \right)\;E_{0},$$

where $E_{0}$ is the incident electric field and M(ω) is the frequency-dependent field enhancement factor associated with the localized surface plasmon resonance.

Because this near-field enhancement amplifies both the excitation field and the Raman-scattered field, the overall SERS intensity scales approximately with the fourth power of the field enhancement [48],

(5) $$I_{\mathrm{SERS}}\propto |M\left(\omega_{\mathrm{exc}}\right){|}^{2}\;|M\left(\omega_{\mathrm{sc}}\right){|}^{2}\;I_{\mathrm{Raman}}\;\approx \;{\left|M\right|}^{4}\;I_{\mathrm{Raman}},$$

which explains why molecules located in plasmonic hotspots, where the electromagnetic field varies sharply over nanometric distances, can produce Raman signals many orders of magnitude stronger than in standard Raman spectroscopy.

The magnitude and spatial distribution of this electromagnetic enhancement strongly depend on the size, shape, and architecture of the plasmonic nanostructure, which determine the localization and intensity of plasmonic hotspots. Nano-particle size and shape are two fundamental parameters governing the performance of plasmonic optical trapping and SERS-based detection [48,49]. The nano-particle size directly influences its polarizability and therefore the magnitude of optical gradient and scattering forces acting on the particle. As particle size decreases, Brownian motion becomes increasingly significant, requiring stronger electromagnetic confinement to achieve stable trapping, while larger particles may experience enhanced scattering forces that can destabilize the trap [33,49]. In addition, size also affects the resonance condition of localized surface plasmons, thereby modifying the efficiency of field enhancement in SERS [28,30].

Beyond size, nano-particle shape plays a critical role in determining the distribution and confinement of electromagnetic fields. Anisotropic or sharp-featured geometries such as rods, tips, or nanogaps can generate highly localized “hot spots” where the electromagnetic field is strongly enhanced, leading to significantly increased SERS signals compared to spherical particles [28,30,48]. Therefore, both size and shape must be carefully engineered to optimize the balance between trapping stability and spectroscopic sensitivity in POT-SERS platforms. In addition, hybrid or hierarchical nano-particle architectures, such as core–shell or multicomponent plasmonic systems, offer an additional strategy to enhance SERS performance by increasing electromagnetic hotspot density and enabling tunable plasmonic coupling [50].

In addition to this electromagnetic enhancement, a secondary contribution known as chemical enhancement can arise from electronic interactions between the adsorbed molecule and the metal surface. These interactions modify the molecular polarizability and may alter resonance conditions, further increasing the Raman response for molecules directly bound to the metal. The combination of electromagnetic and chemical enhancements leads to overall signal amplification factors that can reach $10^{4}$–$10^{10}$, enabling detection down to the single-molecule level under optimized conditions [11,30,35,48,51]. Importantly, SERS provides detailed vibrational fingerprints that reflect molecular composition, bonding configuration, and conformational state, as well as information about the local chemical environment, making it a powerful tool for nanoscale chemical and biological characterization.

## 3. Emerging Plasmonic Architectures for Manipulation and Label-Free Detection

Recent advances in POTs have demonstrated remarkable progress in nano-particle manipulation through structural designs, material selection, and multifunctional capabilities. This subsection highlights advances in nanostructure geometries, force enhancement strategies, and on-chip integration approaches.

### 3.1. Plasmonic Nanostructures for Trapping and Dynamic Manipulation of Nano-Particles

**Figure 2:**
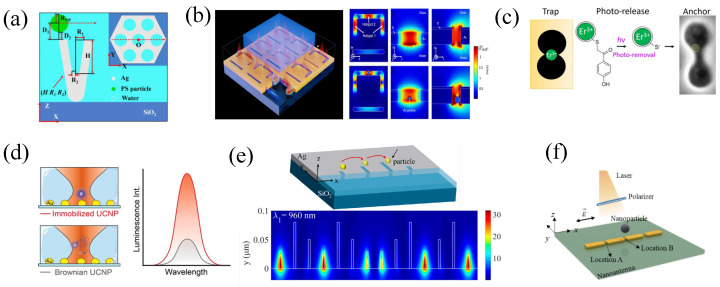
Representative examples of plasmonic nanostructures for nano-particle trapping. (**a**) Ag@PDMS nanocavity array structure for multi-site trapping of polystyrene particles (adapted from [52], Copyright 2024, with permission from AIP Publishing). (**b**) Metamaterial nanostructure showing electric-field distribution for an empty cavity and for a cavity containing a 20 nm gold particle (adapted from [56], Copyright 2023, with permission from American Chemical Society). (**c**) Optical trapping and chemical anchoring of ${\mathrm{NaYF}}_{4}$ nano-particles using DNH (adapted from [57], Copyright 2023, with permission from American Chemical Society). (**d**) Schematic Optical trapping of an upconverting nano-particle using a substrate partially covered with Au plasmonic nanostructures (adapted from [58], Copyright 2024, with permission from American Chemical Society). (**e**) Schematic illustration of the conveyor belt with switchable trapping sites and the electric distribution for the first-order mode (adapted from [60], Copyright 2026, with permission from MDPI). (**f**) Schematic of the nanostructure array for particle sorting (adapted from [49], Copyright 2022, with permission from Optica Publishing Group).

Optimizing nanostructure geometries has been a central focus in POT research to enhance trapping performance. Yan et al. demonstrated stable trapping of polystyrene (PS) particles as small as 14 nm in radius using an Ag@Polydimethylsiloxane nanocavity array at 532 nm excitation with a laser power of 0.9 mW [52]. The hexagonal conical nanocavity array enabled simultaneous activation of multiple trapping sites, a critical requirement for high-throughput assays (Figure 2a). Complementing this work, Nie et al. theoretically investigated bowtie-nanohole tweezers for both trapping and chiral sorting applications [53]. They noted that for chiral particles with opposite chirality parameters, the bowtie-nanohole structure captures one enantiomer while repelling the other when the refractive index of the surrounding solution matches that of the particles, achieving enantioselective separation with a potential well difference reaching 4.8 $k_{B}$T at a wavelength of 700 nm [53]. Further enhancing trapping forces through structural multiplicity, Radman et al. [54] designed a triple-coaxial nano-aperture structure consisting of a 10 nm aluminum oxide insulating layer on a glass substrate, followed by a 65 nm gold film into which three coaxial nano-apertures were fabricated. By optimizing the aperture radius (R = 80 nm for side apertures, $R_{m}$ = 20 nm for the middle aperture) and center-to-center distance (D = 180 nm), they achieved resonant coupling between adjacent apertures. This design produced a transmission coefficient of 0.25 at 795 nm—a 100% enhancement over single apertures—and a twenty-fold enhancement in optical force for 30 nm PS particles compared to single-aperture designs.

Another design was proposed by Ghorbanzadeh, who combined a double nanohole (DNH) with a ${\mathrm{Si}}_{3}$$N_{4}$ tapered waveguide to excite wedge plasmon polariton modes at the nanohole cusps [55]. This approach eliminates bulky free-space optics and enables on-chip integration. Numerical simulations revealed electric-field enhancements exceeding 2700 with sub-2 nm confinement, while comparison of Maxwell stress tensor and point-dipole calculations confirmed a ten-fold force enhancement due to the self-induced back-action (SIBA) effect, allowing trapping with lower light intensity. In addition, Bouloumis et al. demonstrated SIBA-assisted trapping of 20 nm gold particles in Fano-resonant metamaterials, achieving high trap stiffness at 0.61 mW/μ$m^{2}$ [56] (Figure 2b).

The ability to anchor trapped particles has enabled quantum photonics applications. Frencken et al. demonstrated photochemical anchoring of singly ${\mathrm{Er}}^{3+}$-doped nano-particles in gold DNH, creating scalable single-photon emitters at the telecommunications wavelength of 1550 nm [57] (Figure 2c). Fundamental insights into Brownian motion were provided by Zhang et al., who used a glass substrate deposited with 95 nm gold particles (resonant at 574 nm) to trap individual hexagonal ${\mathrm{NaYF}}_{4}$:${\mathrm{Tm}}^{3+}$, ${\mathrm{Yb}}^{3+}$ upconverting nano-particles (43 nm ± 4 nm) using POTs [58]. They revealed that Brownian motion governs plasmonic enhancement, with a time-averaged 20% enhancement extending to 1 µm rather than the six-fold enhancement predicted for static nano-particles at sub-80 nm distances [58] (Figure 2d). A practical method for monitoring local temperature in trapping experiments was developed by Toodeshki and coworkers, who used Er-containing ${\mathrm{NaYF}}_{4}$ nanocrystals (20 nm diameter) trapped in DNH [59]. By performing ratiometric analysis of the emission at 527 nm and 542 nm, they measured the local temperature rise directly using the trapping laser itself, eliminating the need for a separate excitation source. They found that thicker gold films substantially reduce heating: 70 nm films give 0.64 K/mW, 100 nm give 0.37 K/mW, and 130 nm give 0.25 K/mW.

Dynamic manipulation represents a critical capability for practical applications. Wang et al. designed a plasmonic multi-slot array with three resonant wavelengths enabling stepwise transport of 20 nm particles with 70 nm displacement per step, allowing precise control of inter-particle distance [60] (Figure 2e). Remarkably, Qin et al. integrated a gold cross-antenna tweezer into a light-driven microdrone, creating a mobile microrobot that traps, transports, releases, and recaptures single 70 nm diamonds. The device achieves linear translation at 4.55 μm/s and complete trap–transport–release sequences via helicity modulation [61]. Given that heating is unavoidable in plasmonic structures, understanding photothermal effects is essential. Ji et al. quantified that photothermal effects reduce capture efficiency by nearly 25%, with thermophoretic forces inhibiting trapping while thermo-osmotic forces assist capture [62]. Importantly, when substrate thermal conductivity reaches 20 W/(m·K), photothermal effects become negligible, providing a clear design guideline for thermal management.

Particle sorting adds another dimension to POT utility. Li et al. designed a gold nanoarray with graded gaps achieving 97% capture of high-index particles at the first gap, enabling label-free continuous sorting [49] (Figure 2f). For asymmetric particles, Koya et al. achieved three-fold force enhancement for Janus nano-particles using dual-laser resonant trapping, with orientation-dependent trapping critical for nanorobotics [63]. Addressing the trade-off between confinement and heating, Colapietro et al. [64] proposed a hybrid dielectric–plasmonic nanobowtie achieving over 90% energy confinement improvement while trapping 100 nm virus-sized particles with stability exceeding ten times the thermal energy and temperatures remaining below virus inactivation thresholds.

A challenge in POT is the diffusion limitation, where particles randomly diffuse to within tens of nanometers of the hotspot before trapping can occur, a process that can take minutes for dilute samples. Anyika and coworkers addressed this by combining DNH with an applied AC field to induce electrothermoplasmonic flow [65]. Using a 10 V, 3 kHz AC bias and a 973 nm trapping laser at 11.4 mW/μ$m^{2}$, they achieved rapid transport of a 25 nm PS particle across 63 μm and trapping within 16 s [65]. The continuous gold film efficiently dissipated heat, while the synergistic combination of optical and thermophoretic forces produced a total trapping potential of $-3.9k_{B}T$, sufficient for stable trapping. This platform shows great promise for applications requiring simultaneous trapping and plasmon-enhanced spectroscopies, such as SERS.

#### Trapping and Sensing of Biological Entities

**Figure 3:**
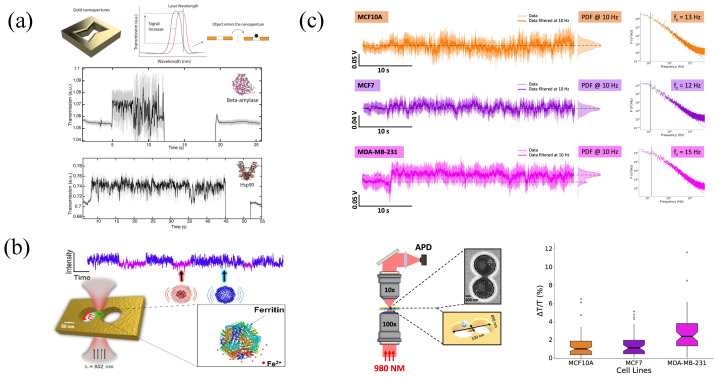
Optical trapping of single proteins and extracellular vesicles using double nanohole (DNH) apertures. (**a**) Transmission signal of a single β-amylase protein trapped in an inverted bowtie nano-aperture, showing a sudden increase upon trapping and a return to baseline after the laser is turned off (adapted from [66], Copyright 2021, with permission from Elsevier). (**b**) Transmission signal of a single holo-ferritin protein trapped in a DNH, exhibiting reduced fluctuations compared to apo-ferritin due to its more rigid conformation (adapted from [69], Copyright 2023, with permission from American Chemical Society). (**c**) Representative trapping signals of single extracellular vesicles derived from non-malignant (MCF10A), non-invasive cancerous (MCF7), and invasive cancerous (MDA-MB-231) cell lines, showing distinct transmission characteristics for each EV type (adapted from [71], Copyright 2024, with permission from IOP Publishing).

The ability to trap and manipulate individual biological entities at the nanoscale has profound implications for diagnostics, drug discovery, and fundamental biology. POTs have emerged as powerful tools for this purpose, offering non-contact manipulation with high spatial resolution and low laser powers that preserve biological photodamage. A demonstration in the field was the long-term trapping of unmodified proteins using inverted bowtie nano-apertures [66]. Yang and colleagues fabricated arrays of inverted bowtie-shaped plasmonic gold nanopores with gap sizes of 10–20 nm using focused ion beam milling (FIB) [66] (Figure 3a). They successfully trapped single β-amylase and Heat Shock Protein 90 (HSP90) for extended periods exceeding three minutes, with trapping events noted by an increase in transmission signal due to the red-shift of the plasmonic resonance upon protein entry. The authors observed distinct fluctuation levels in the trapping signal, suggesting different conformational states of the trapped proteins [66].

The energy landscape governing protein conformational changes was directly resolved by Peters and coworkers using DNH optical tweezers with bovine serum albumin (BSA) as a model system [67]. By deconvolving the measured transmission signal with a Gaussian point spread function, they extracted the probability density function, which represents free energy landscape of BSA, revealing three distinct states: native (N), fast (F), and expanded (E). Importantly, the authors demonstrated that the extracted energy landscape agrees quantitatively with kinetic residence time analysis, validating the deconvolution approach for single unmodified proteins [67]. For enzyme dynamics, Kotsifaki et al. employed metamaterial optical tweezers to trap single urease molecules with a hydrodynamic radius of 7.4 nm [68]. By introducing ${\mathrm{PEG}}_{6000}$ as a crowding agent, they exploited the synergy between thermophoretic and depletion forces to achieve trapping at remarkably low laser intensities of 0.13 mW/μ$m^{2}$. The structural dynamics of ferritin—a key iron storage protein—were investigated by Yousefi et al., using DNH optical tweezers [69]. By trapping individual apo-ferritin (iron-free) and holo-ferritin (iron-loaded) proteins, they observed that holo-ferritin produced a lower root-mean-square deviation in the transmission signal compared to its apo counterpart, consistent with the more rigid, compact conformation of iron-loaded ferritin [69] (Figure 3b). In a subsequent study, the same group monitored the in situ iron loading of a single apo-ferritin molecule in real-time [70]. Upon exposure to ${\mathrm{Fe}}^{2+}$ solution, the trapping signal exhibited distinct “on–off” patterns with dwell times of 2–5 s, attributed to the cyclic gating of the threefold channels: unfolding to allow ${\mathrm{Fe}}^{2+}$ entry to ferroxidase centers, followed by folding to transfer ${\mathrm{Fe}}^{3+}$ to the protein core [70]. After approximately 20–30 min, the signal amplitude decreased with reduced fluctuations, indicating successful iron mineralization.

Small extracellular vesicles (sEVs), ranging from 30 to 150 nm in diameter, have emerged as promising biomarkers for cancer diagnostics due to their abundance in biological fluids and their cargo of lipids, proteins, and nucleic acids from parent cells. However, the inherent heterogeneity of sEVs populations poses significant challenges for accurate detection, motivating the development of single-entity trapping platforms. Peters and colleagues demonstrated a classification of single sEVs using DNH optical tweezers combined with ML [71]. Trapping signals from sEVs derived from three cell lines—non-malignant MCF10A, non-invasive cancerous MCF7, and invasive cancerous MDA-MB-231—were recorded and analyzed using a one-dimensional convolutional neural network (1D CNN) (Figure 3c). The classifier achieved 88% overall accuracy in distinguishing the three sEVs types, with the invasive cancerous sEVs (MDA-MB-231) showing a distinct stronger initial trapping step and different power spectral density characteristics compared to non-cancerous sEVs [71]. Critically, the specificity for identifying cancerous versus non-cancerous sEVs reached 98%, demonstrating the clinical potential of this label-free approach.

Hong and colleagues introduced Interferometric Electrohydrodynamic Tweezers (IETs), a platform that integrates rapid parallel trapping, label-free interferometric imaging, and Raman spectroscopy for comprehensive single-particle analysis [72]. The device uses a 15 nm-thick gold film patterned with an array of microscale holes (12,321 trapping sites). Upon application of AC voltage, the electro-osmotic flows transport nano-particles to stagnation zones at the center of four adjacent holes, achieving trapping within seconds [72]. The platform operates in transmission mode, collecting forward-scattered light that interferes with transmitted light to produce interferometric contrast images. Unlike reflection-mode iSCAT, where contrast scales nonlinearly with particle size, the forward-scattering geometry yields a nearly linear relationship between contrast and particle size up to approximately 300 nm, enabling direct size estimation. The platform trapped small sEVs with estimated radii of 91 nm, 64 nm, and 38 nm, confirmed by Brownian diffusion analysis. Raman spectroscopy of individual trapped sEVs revealed characteristic peaks at 876 ${\mathrm{cm}}^{-1}$ (proteins), 977 ${\mathrm{cm}}^{-1}$ (proteins), 1088 ${\mathrm{cm}}^{-1}$ (nucleic acids/lipids/proteins), 1281 ${\mathrm{cm}}^{-1}$ (amide III), 1454 ${\mathrm{cm}}^{-1}$ (${\mathrm{CH}}_{2}$/${\mathrm{CH}}_{3}$ modes), and 1665 ${\mathrm{cm}}^{-1}$ (amide I), establishing the ability to profile the global bio-molecular composition of single sEVs in their native state. Addressing the challenge of photothermal destabilization in POT, Anyika and coworkers developed a hybrid approach combining plasmonic heating with diffusiophoretic forces [73]. Using resonant bowtie nano-antennas in a 12% polyethylene glycol (${\mathrm{PEG}}_{8000}$) solution, laser illumination at 973 nm generated localized temperature gradients that thermophoretically depleted PEG molecules from the hotspot, creating sharp concentration gradients. These gradients induced an attractive diffusiophoretic force that overcame the repulsive thermophoretic force. The authors demonstrated stable trapping of 100 nm PS beads at laser powers as low as 0.78 mW, with trap stiffness decreasing with increasing power—a result attributed to the sign reversal of the effective Soret coefficient at higher temperatures. Importantly, they trapped sEVs at 1 mW where the temperature rise of 13.3 K kept the sample below 37 °C, preventing photodamage [73].

The architectures described in Table 1 illustrate a clear trade-off between trapping performance, throughput, and fabrication complexity. Nanocavity arrays offer multi-site trapping at low power but exhibit limited tunability [52]. Bowtie and DNH designs provide strong near-field confinement but suffer from low throughput and may require precise nanofabrication [53,54,55]. Metamaterial and hybrid approaches improve trapping stiffness but introduce additional complexity [56]. Significantly, the field currently lacks a systematic comparison of these architectures under identical conditions, making it difficult to identify which design is optimal for specific applications. For biological trapping, the hybrid dielectric–plasmonic nanobowtie appears particularly promising because it addresses the critical challenge of photothermal damage [64].

### 3.2. Integrated Platforms: Simultaneous Manipulation and Chemical Interrogation

Conventional SERS averages signals from large populations, hiding molecular heterogeneity, while standard optical tweezers provide positional control but lack chemical fingerprinting capability. Integrated trapping-SERS platforms overcome both limitations by using the same plasmonic near-field to confine a single particle at a hotspot and record its Raman spectrum. Tiwari et al. [74] demonstrated that a single 100 nm gold particle illuminated at 633 nm (0.1 mW/μ$m^{2}$) generates a temperature gradient driving thermophoretic and convective flows, capturing nearby nano-particles and forming reversible assemblies at the trap site. Using bianalyte SERS statistics (BPE and 2-NAT), they confirmed single-molecule sensitivity, demonstrating both trapping and directed assembly while recording Raman spectra. Ma et al. [75] used a bull’s eye nanostructure—a circular grating milled into a gold film that focuses surface plasmons to a central hotspot—to trap a single 5 μm PS bead or living yeast cell at 8 mW (785 nm). By measuring both trap stiffness (via power spectral density) and SERS intensity simultaneously, they revealed that SERS intensity scales linearly with trap stiffness, providing a calibration framework linking mechanical trapping to electromagnetic enhancement. Fu et al. [76], constructed a dynamic plasmonic nanocavity for single-molecule protein analysis without surface immobilization—avoiding the denaturation that plagues conventional SERS. Two 3 μm silica beads coated with sparse 50 nm silver particles were brought together using a 1064 nm optical trap (8.0 mW), while a freely diffusing 80 nm silver particle was trapped at their junction, creating a 20 nm gap. A separate 532 nm laser (2.7 mW) excited SERS with $10^{9}$ enhancement, sufficient for single-molecule detection. Using this platform, they detected pH-dependent secondary structure changes in hIAPP: α-helical at pH 7.4 versus β-sheet at pH 5.5—the conformation linked to Type 2 diabetes [76].

Khosravi and Gordon demonstrated the combination of DNH optical trapping with Raman spectroscopy for chemical characterization of single nano-particles using a commercial Raman system [17]. For 21 nm titania (Ti$O_{2}$) particles, the trapped particles produced Raman spectra with four characteristic peaks corresponding to anatase molecular vibrations ($E_{g}$, $B_{1g}$), achieving 400 counts/s with only 14 mW of laser power [17]. In addition, they observed that the Raman intensity at 145 ${\mathrm{cm}}^{-1}$ increased as the DNH gap size decreased, confirming the plasmonic enhancement mechanism. Additionally, Zhang and coworkers developed TrapNet, a more sophisticated deep learning architecture incorporating four convolutional layers followed by a Kolmogorov–Arnold network (KAN) layer for enhanced nonlinear feature extraction [77]. Using an expanded dataset of 502 independent trapping sessions across multiple days, operators, and sEVs batches, TrapNet achieved 99% overall accuracy in classifying MCF10A, MCF7, and MDA-MB-231 sEVs, with perfect 100% accuracy in distinguishing cancerous from non-cancerous samples. Analysis of the latent feature space via t-distributed stochastic neighbor embedding (t-SNE) revealed well-defined clusters corresponding to each sEVs type, with the mean power spectral density showing a clear gradient from cancerous (lowest) to non-cancerous (highest). The authors attributed these differences to underlying biophysical properties including size, shape, refractive index, and surface charge, which correlate with trap stiffness [77]. Notably, TrapNet demonstrated robustness across domain shifts—variations caused by different operators, sEVs batches, and DNH geometries—whereas conventional models like LSTM, Bi-LSTM, and Transformer showed overlapping clusters and reduced generalization.

Integrated POT-SERS platforms are still at an early stage of development, with most studies focusing on either optical trapping or SERS, while only a limited number successfully integrate both functionalities within a single platform (Table 2). The dynamic plasmonic nanocavity achieves the highest SERS enhancement ($10^{9}$) by creating a tunable hotspot using dual optical traps [76], while the DNH Raman tweezer offers a simpler, commercially accessible approach with a moderate enhancement [17]. The IET platform enables massively parallel trapping [75]. A significant gap exists in the integration of real-time SERS feedback with active trapping control, representing a priority for future development.

## 4. The Role of Artificial Intelligence in Plasmonic Nanophotonics

**Figure 4:**
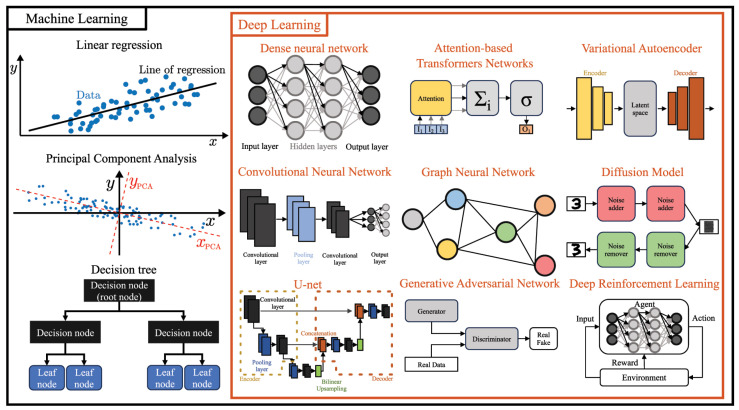
DL (orange rectangle) is a specialized subset within the broader field of ML (black rectangle). Machine learning methods include techniques such as linear regression, principal component analysis, and decision trees. Deep learning methods encompass dense neural networks, convolutional neural networks, U-Nets, attention-based transformer networks, graph neural networks, generative adversarial networks, variational autoencoders, diffusion models, and deep reinforcement learning (adapted from [25], Copyright 2024, with permission from Wiley).

Data analysis in SERS and plasmonic optical trapping is challenged by the complexity and variability of plasmon-enhanced signals [78,79]. In SERS, low signal-to-noise ratios, spectral overlap, and nonlinear interactions in complex mixtures produce crowded vibrational spectra that complicate compound identification. In addition, surface selection rules and differential signal enhancement distort relative peak intensities across compounds [79]. In POT, Brownian motion, photothermal effects, and temporal fluctuations in the optical-force landscape influence particle localization, trap stiffness, and residence time, reducing measurement reproducibility [29,80]. Traditional analytical approaches often depend on manually engineered features and predefined assumptions [25,81]. However, ML methods can learn patterns and relationships directly from data, making them well suited for high-dimensional, noisy plasmonic datasets [82] (Figure 4). By modeling complex nonlinear relationships, ML improves the extraction of robust and reproducible features under experimental variability. DL, a subset of ML based on multilayer neural networks, further extends these capabilities by automatically learning hierarchical representations from raw or minimally processed spectra, reducing the need for manual feature extraction while maintaining subtle diagnostically relevant information [24,82,83]. In addition, DL enables end-to-end learning, in which feature extraction and prediction are jointly optimized within a unified model, reducing error propagation across processing steps and improving robustness to noise and experimental variability [84] (Figure 4). These characteristics make ML well-suited for plasmon-enhanced data analysis.

### 4.1. Decoding SERS Spectra with Machine Learning Techniques

**Figure 5:**
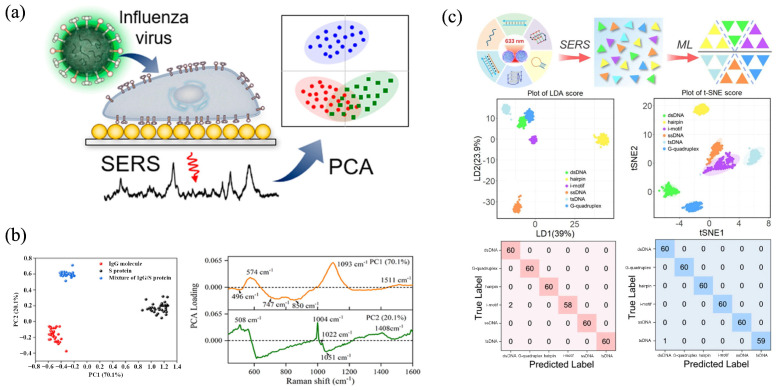
(**a**) Schematic of identification of cells infected with influenza viruses, where the type of viruses was extracted via PCA of Raman signatures (adapted from [86], Copyright 2019, with permission from ACS Publications). (**b**) PCA score plot of SERS spectra for spike protein (black), IgG antibodies (red), and their mixture (blue). PC1 and PC2 account for 70.1% and 20.1% of the variance, respectively. The loading plot shows the Raman shifts that contribute most to each principal component (adapted from [20], Copyright 2025, with permission from Wiley). (**c**) Linear discriminant analysis (LDA) and t-distributed stochastic neighbor embedding (t-SNE) projections of SERS spectra for six DNA types, along with confusion matrices showing classification results for unknown DNA samples using LDA and support vector machines (adapted from [89], Copyright 2024, with permission from ACS Publications).

SERS combined with ML has been applied to analyze cancer-derived exosomes [85]. The heterogeneity of SERS signals makes it difficult to identify cancer-specific spectral features using conventional methods. Principal component analysis (PCA), an unsupervised ML technique, addresses this challenge by reducing high-dimensional spectral data into dominant patterns. The first principal component accounted for 61.7% of the total variance in the dataset, and its loading vectors revealed 26 candidate Raman peaks that distinguished cancerous exosomes from normal ones [85]. A subset of 13 of these features showed a linear correlation with cancer exosome concentration exceeding 90%. These ML-derived spectral fingerprints were then compared against Raman profiles of known protein markers using Euclidean distance. Among the markers examined, EGFR showed the highest similarity to the cancer-associated spectral patterns, which was validated by immunoblotting [85]. For influenza virus detection, viral envelope proteins displayed on infected cell surfaces generate SERS signals, but cellular proteins produce overlapping spectral features that complicate direct identification (Figure 5a) [86]. PCA resolves this issue by reducing high-dimensional spectral data into principal components that maximize variance between sample groups. In one implementation, the first two principal components captured the dominant spectral differences, and 95% confidence ellipses in the PCA score plots completely separated infected from uninfected cells with statistical significance [86]. Key Raman shifts at 737, 1331, 1467, and 1625 ${\mathrm{cm}}^{-1}$ were identified as characteristic of WSN strain infection, while shifts at 609, 720, and 1625 ${\mathrm{cm}}^{-1}$ distinguished CAL strain infection. The method also discriminated cells infected with a genome-assorted mutant virus expressing WSN hemagglutinin and CAL neuraminidase from both parental strains [86]. PCA further distinguished cells expressing only hemagglutinin from those expressing only neuraminidase, with characteristic peaks at 1206 ${\mathrm{cm}}^{-1}$ for CAL hemagglutinin and at 740 and 859 ${\mathrm{cm}}^{-1}$ for CAL neuraminidase [86]. PCA has also been applied to SERS data for the detection and discrimination of SARS-CoV-2 and its associated biomarkers [20,87,88]. Using a gold film substrate, SERS spectra were collected from four coronaviruses, including SARS-CoV-2 and the Omicron BA.5 variant, with limits of detection below 100 ${\mathrm{TCID}}_{50}$/mL. The first three principal components captured over 80% of the spectral variance, enabling clear separation of all four virus types in three-dimensional PCA space [87]. In another study using an array of *H*-shaped nano-apertures, PCA distinguished SARS-CoV-2 spike protein from IgG antibodies and their mixture, with the first two principal components accounting for 90.2% of the total variance (Figure 5b) [20]. A direct comparison between empirical peak assignment and ML was performed using clinical nasopharyngeal swab samples from patients infected with the BA.2 variant [88]. An ensemble subspace k-nearest neighbors model trained on 394 spectra achieved a sensitivity of 85.7% and a specificity of 60% on a blind test set, outperforming manual peak assignment, which yielded a specificity of only 40% [88]. For DNA analysis, a SERS platform using thiol-modified silver nano-particles eliminated citrate interference and enabled direct detection of six DNA conformations, including single-stranded, double-stranded, i-motif, hairpin, G-quadruplex, and triple-stranded DNA (Figure 5c) [89]. Unsupervised learning methods, including linear discriminant analysis and t-distributed stochastic neighbor embedding, projected the high-dimensional SERS data into two-dimensional space, where all six DNA types formed distinct, well-separated clusters. The contribution rates of the first two linear discriminant components were 39% and 23.9%, respectively [89]. Supervised learning algorithms, including linear discriminant analysis and support vector machines, achieved prediction accuracies exceeding 99% for DNA classification [89]. For circulating tumor cell detection, dual-modal SERS bioprobes were designed with two Raman reporter molecules and combined with PCA and a random forest classifier [90]. PCA reduced the spectral dimensionality, and the random forest model achieved a detection rate of 98% for distinguishing tumor cells from white blood cells, with a four-class classification accuracy of 90% across three tumor cell lines and white blood cells [90]. The limit of detection was 2 cells per milliliter in simulated blood samples. The abovementioned studies demonstrate that ML enables consistent and rapid classification of complex clinical SERS data, while empirical methods provide biochemical interpretability.

DL methods have become increasingly prominent. For example, a variational autoencoder was applied to encode SERS spectra of bacterial lysate from Pseudomonas aeruginosa and *Escherichia coli* [91]. The encoded spectra formed clear clusters separating untreated bacteria from those exposed to antibiotics. A deep neural network (DNN) then differentiated metabolic responses as early as 10 min after antibiotic exposure with greater than 99% accuracy, at concentrations up to ten-fold lower than the minimum inhibitory concentration determined by conventional growth assays [91]. The generative nature of the variational autoencoder also enabled identification of spectral features associated with antibiotic efficacy, specifically in the 1100 ${\mathrm{cm}}^{-1}$ to 1200 ${\mathrm{cm}}^{-1}$ region corresponding to aromatic ring vibrations [91]. Based on this insight, a dataset of six aromatic metabolites was selected, and unsupervised Bayesian Gaussian mixture analysis achieved 99.3% classification accuracy in discriminating susceptible from resistant antibiotic responses using only a single spectrum per category [91].

Convolutional neural networks (CNNs) are particularly effective in extracting local spectral features and capturing characteristic peak patterns, making them well-suited for label-free disease classification tasks. A one-dimensional CNN was subsequently developed for cancer detection using label-free serum SERS spectra from healthy controls and patients with bladder cancer, adrenal cancer, and acute myeloid leukemia [92]. After spectral data augmentation, the 1D-CNN achieved a classification accuracy of 98.27% across the four groups [92]. Gradient-weighted class activation mapping was then used to interpret the model’s decisions, revealing that L-tyrosine was the most important biomarker for bladder cancer, acetoacetate and riboflavin for adrenal cancer, and phospholipids, amide-I, and α-helix for acute myeloid leukemia [92]. A residual neural network-based DL model was then built to screen small-molecule binding sites in proteins [93]. A SERS dataset comprising the small molecules fomepizole and ibrutinib, along with other molecules, was used to train a ResNet model, which achieved classification accuracy exceeding 99% [93]. The model then processed SERS spectra from high-performance liquid chromatography (HPLC) fractions of proteolytic digests of small-molecule-protein complexes, generating a probability map that identified fractions containing small-molecule-modified peptides [93]. Subsequent mass spectrometry analysis of these target fractions successfully identified fomepizole binding sites coordinated with zinc in alcohol dehydrogenase and ibrutinib binding at cysteine residues in Bruton tyrosine kinase.

Single-molecule discrimination of proline and hydroxyproline was achieved using a particle-in-pore SERS sensor combined with a 1D-CCN [94]. The occurrence frequency histogram of single-molecule SERS peaks was used to filter out intensity fluctuations while preserving structural information. Citrate interference was minimized by incubating gold nano-particles in the analyte solution for 48 h to achieve monolayer coverage, after which citrate bands became nearly negligible [94]. The 1D-CNN model then distinguished proline from hydroxyproline with 96.6% accuracy. Gradient-weighted positive feature visualization confirmed that the model’s discrimination relied on Raman bands corresponding to hydroxylation-induced changes in the pyrrolidine ring [94].

Gold nanostars with strong SERS enhancement were synthesized and used as nanoprobes for influenza B detection [95]. A large-area Raman scanning strategy generated spatial intensity maps of immunoprobe distribution along the test line, converting point-based measurements into image data. A ResNet-18 model analyzed these SERS images and achieved 100% training accuracy and 95% validation accuracy, substantially outperforming conventional peak intensity analysis and support vector machine-based full-spectrum discrimination [95]. The system was then adapted for influenza A detection by changing the capture and detection antibodies, and testing on 40 simulated human clinical samples yielded 95% accuracy.

### 4.2. Data-Driven Design of Plasmonic Nanostructures

The traditional design for plasmonic nanostructures relies on iterative forward simulations—typically using finite-difference time-domain (FDTD) or finite element methods (FEM)—where the researcher guesses a geometry, simulates its optical response, and manually refines the parameters. This approach becomes time-consuming as the dimensionality of the design space increases, and it may fail to address the inverse problem: obtaining a geometry that produces a desired electromagnetic response. DL has emerged as a transformative solution to this challenge. Unlike conventional optimization algorithms that search the parameter space for each design task individually, a neural network learns the underlying mapping between geometry and optical response once, after which inverse design reduces to a simple forward pass through the network—a process that takes milliseconds rather than hours or days.

**Figure 6:**
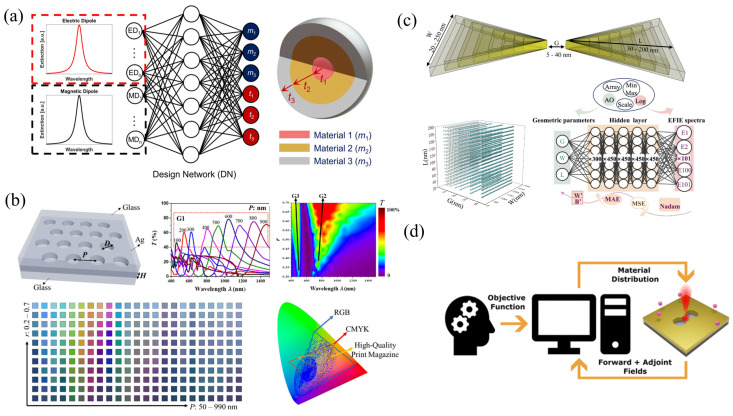
(**a**) Schematic illustrations of a core–shell nano-particle, and the dual-neural-network deep learning model employed in this study, which comprises a design network (DN) and a simulation network (SN). The DN learns the mapping from the input extinction spectra (*S*) to the design parameters (*D*), while the SN learns the mapping from the design parameters (*D*) back to the extinction spectra ($S^{\prime }$) (adapted from [96], Copyright 2019, with permission from ACS Publications). (**b**) Schematic illustration of a silver nanoarray (Ag NAs). Representative transmission spectra T(λ) of Ag NAs as the period *P* increases from 100 to 900 nm, while the ratio r=D/P is fixed at 0.5. The peaks within the dotted rectangle are labeled G1, with the corresponding *P* value indicated above each peak. Color map of T(λ) as a function of *r* at a fixed P=400 nm. The two observed peaks are denoted G2 and G3, respectively. Color palette obtained by varying both *P* and *r* while keeping the height *H* constant at 50 nm. CIE 1931 xy chromaticity coordinates derived from the calculated T(λ) spectra. The blue, red, and yellow triangles mark the color gamut boundaries of standard RGB (sRGB), CMYK, and high-quality print magazine color space, respectively (adapted from [97], Copyright 2023, with permission from Wiley). (**c**) Bowtie nano-antenna geometry with variable prism width (*W*), prism length (*L*), and gap (*G*) between prism tips, and a fixed thickness of 50 nm. Distribution of 3024 groups of training data collected by FDTD. Optimized DNN for predicting and designing the near-field EFIE (${\left|E\right|}^{2}$) with adaptive methods such as data preprocessing and cost function selection (adapted from [98], Copyright 2021, with permission from OPTICA). (**d**) Topology optimization for inverse design of plasmonic nano-apertures. The optimized structure enhances trapping potential compared to conventional forward designs (adapted from [99], Copyright 2023, with permission from ACS Publications).

For instance, Malkiel et al. provided a demonstration of this paradigm using a bidirectional deep neural network (DNN) for plasmonic nanostructures patterned as general *H*-shaped meta-atoms [26]. The model consisted of two jointly trained modules, one dedicated to geometry prediction and the other to spectrum prediction. Once trained on more than 15,000 synthetic experiments generated via COMSOL Multiphysics 4.3b simulations, the DNN could retrieve subwavelength dimensions from far-field transmission spectra alone. Experimental validation on fabricated gold nanostructures showed excellent agreement between DNN-predicted geometries and measured spectra, with the network even generalizing to boundary cases that it had never seen during training [26]. So et al. extended this approach to simultaneous inverse design of both materials and structural parameters in core–shell nano-particles (Figure 6a) [96]. Their key innovation was a loss function that combined regression for continuous thickness parameters and classification for discrete material choices, indexed from seven possibilities including Ag, Au, Al, Cu, Ti$O_{2}$, Si$O_{2}$, and Si. The bidirectional network took as input the extinction spectra of electric and magnetic dipoles separately, enabling the direct engineering of multipolar responses [96]. The trained network could spectrally tune electric dipole resonances, find spectrally isolated magnetic dipole modes, and achieve spectrally overlapped electric and magnetic dipole resonances for directional scattering or negative-index media. Once trained, the network provided structural designs within one second.

A fundamental challenge in inverse design is the “one-to-many” mapping problem: multiple distinct nanostructures can produce nearly identical optical responses. Bidirectional tandem networks address this by training forward and inverse networks simultaneously, using the forward network to constrain the inverse predictions. Liu et al. extended this concept by incorporating structural color alongside spectral data for silver nanohole arrays (Figure 6b) [97]. They trained a spectrum–structure–color network that took transmission spectra as input and output both structural parameters (period and hole diameter) and RGB color coordinates. Training on color proved more efficient than training on the full 354-point spectrum, reducing training time while achieving high accuracy [97]. The inverse-designed nanohole arrays could then be used to regenerate full-color images, with the entire reconstruction completed in seconds. Wu et al. addressed a different data challenge: predicting near-field intensity enhancement, which spans orders of magnitude from single digits to over 60,000 (Figure 6c) [98]. Using logarithm preprocessing and mean absolute error loss rather than standard mean squared error, their DNN accurately predicted both near-field enhancement and far-field transmission spectra simultaneously for bowtie nano-antennas. The inverse-designed nano-antennas achieved electric-field enhancements up to 89,883—one to two orders of magnitude higher than conventional designs—demonstrating that deep learning can discover structures that outperform human intuition [98].

A criticism of DL approaches is their “black box” nature. Yeung et al. addressed this using explainable AI methods, specifically Deep SHapley Additive explanations, to identify the spatial regions of nanophotonic structures that most strongly influence absorption spectra [100]. They trained a CNN on 10,000 metal-insulator-metal metamaterial resonators to predict mid-infrared absorption spectra from geometry images. The CNN achieved over 95% mean absolute accuracy and was 6,500 times faster than full FDTD simulations [100]. Using Deep SHAP, they generated pixel-by-pixel heatmaps showing which structural features contributed positively or negatively to absorption at specific wavelengths. For simple cross-shaped resonators, the heatmaps revealed that the CNN had learned the physical relationship between arm length and resonance wavelength. For complex freeform structures, the heatmaps identified which concave or protruding features controlled specific absorption peaks [100].

Nelson et al. applied topology optimization—an inverse design method distinct from DL—to plasmonic nanotweezers (Figure 6d) [99]. Using adjoint sensitivity analysis, their algorithm produced a central aperture reminiscent of the DNH design and additionally generated nonintuitive outer structures that directed surface plasmon polaritons into the central aperture [99]. These outer structures increased the electric-field intensity by a factor of 4.97, resulting in a trapping potential 1.95 times greater than previous algorithm-designed nanotweezers and 27.9 times greater than conventional double nanohole designs [99].

### 4.3. Artificial Intelligence as an Enabling Technology for Plasmonic Optical Tweezers

Despite rapid progress in applying DL to optical tweezers, most existing studies focus on conventional free-space trapping configurations, where the optical field is comparatively stable and well characterized [25]. On the other hand, POT operates in strongly confined near-field environments, where nanoscale field gradients, surface-induced perturbations, and sensitivity to local geometry make the trapping potential highly nonlinear and difficult to model analytically. As a result, in POT systems AI is not only a post-processing tool but can also serve as an enabling framework for inference, optimization, and real-time control in regimes where first-principles modeling is incomplete.

Within this context, emerging studies suggest pathways toward more direct AI relevance in plasmonic and vortex-assisted trapping. For example, Shen and Liu demonstrated a bidirectional neural network for analyzing and designing optical vortex tweezers, and further validated it using experimental data from plasmonic vortex trapping, showing that learned representations can capture features of plasmonic trapping dynamics [101]. Although still indirect, such results indicate the potential of data-driven models to bridge conventional optical vortex systems and plasmonic trapping configurations.

For the design of plasmonic trapping nanostructures, ML and inverse design approaches have demonstrated significant potential in addressing the inherently complex and highly electromagnetic optimization landscape of POT systems [102]. In particular, simulated annealing and related global optimization strategies have been employed to design plasmonic nano-apertures with irregular geometries, achieving substantially enhanced trapping potentials compared to conventional double nanohole architectures [99,102]. More broadly, recent reviews on inverse design in nanotweezers highlight that computational approaches, including topology optimization, adjoint-based optimization, and ML, enable the discovery of non-intuitive nanostructures that tailor near-field trapping landscapes and enhance trap stiffness in plasmonic configurations [102]. These methods are particularly valuable in POT, where the strong sensitivity of near-field distributions to nanoscale geometry makes analytical design approaches insufficient.

Furthermore, reinforcement learning and closed-loop DL frameworks may provide a natural route toward autonomous plasmonic optical trapping, enabling real-time adjustment of laser parameters, stabilization of nano-particles in fluctuating hotspots, and compensation for thermal and mechanical drift [103]. Together, these developments suggest that AI may evolve from a data analysis tool into a core component of adaptive and autonomous plasmonic nanophotonic systems.

In addition, AI applications in POT-SERS are dominated by supervised learning for spectral classification, with reinforcement learning and autonomous control still in early stages (Table 3). While DL has revolutionized SERS spectral analysis, its application to active POT control remains limited. Deep reinforcement learning has demonstrated success in conventional optical tweezers for autonomous navigation and cell isolation, but its adaptation to plasmonic trapping—steep field gradients, photothermal effects, and nanoscale positioning—remains an open challenge. The primary bottleneck is not algorithmic but experimental: the low throughput of POT experiments makes it difficult to generate the large, labeled datasets required for training robust DL models. Future progress will depend on the development of transfer learning and physics-informed neural networks that can operate with limited experimental data, as well as the integration of simulation environments for training reinforcement learning agents.

## 5. Toward Intelligent Integration: Autonomous Plasmonic Platforms

The transition from manually operated POT-SERS systems to autonomous platforms may require the integration of three functional components: simultaneous multi-modal sensing, real-time data processing using ML, and hardware actuation based on processed spectral and positional information. Current POT-SERS configurations typically perform trapping and spectral acquisition sequentially, with data analysis conducted offline. This open-loop operation limits throughput, introduces operator bias, and prevents adaptive experimentation. The first requirement for closed-loop operation is the ability to measure both the spatial position and the molecular identity of a trapped particle. Dual-trap Raman configurations have demonstrated this feasibility by manipulating nano-particle probes while simultaneously focusing excitation light into the hotspot [105]. Within this configuration, the particle’s position can be tracked using bright-field or dark-field microscopy, while the SERS channel reports its molecular composition. Dark-field microscopy provides sub-diffraction spatial localization and has been successfully combined with SERS to characterize submicron aerosol particles, resolving heterogeneous chemical compositions while mapping spatial positions [106]. For intracellular applications, organelle-targeting gold nanorods have enabled simultaneous SERS acquisition from specific subcellular compartments including the nucleus, mitochondria, and lysosome [107,108]. These studies establish that multi-modal sensing—combining positional tracking with vibrational spectroscopy—is technically feasible within the same nanoscale volume.

The second requirement is real-time data processing. SERS spectra from biological samples are complex, containing overlapping peaks from multiple molecular species, baseline fluctuations, and stochastic intensity variations. Manual analysis is insufficient for closed-loop operation. Convolutional neural networks (CNNs) have been applied to classify SERS spectra of structurally similar molecules with high accuracy. A multilayer perceptron model trained on SERS spectra of adenosine phosphates (AMP, ADP, and ATP) achieved classification accuracy of 91.4% after feature selection and data augmentation, demonstrating that ML can resolve spectral differences among molecules with nearly identical structures [109]. In stem cell differentiation monitoring, a CNN trained on over 6000 SERS spectra from mitochondria-targeted probes achieved 98.5% accuracy in identifying six distinct cell states, including embryoid body stages, across the differentiation process from induced pluripotent stem cells to neural progenitor cells [110]. These results confirm that DL can extract meaningful features from SERS data without manual preprocessing and can do so with sufficient speed and accuracy for real-time applications.

The third requirement is automated hardware actuation based on processed information. The AI-RACS system integrates a microfluidic chip with DL-based object detection, optical tweezers, and automated single-cell collection [111]. The system uses a CNN to identify target cells in real-time from bright-field images, then employs optical tweezers to capture and sort individual microbial cells based on their Raman spectra. The authors demonstrated sorting of aluminum-tolerant microorganisms from acidic soil microbiota, successfully culturing 13 strains including *Burkholderia* spp., *Rhodanobacter* spp., and *Bacillus* spp. All 13 cultured strains showed strong aluminum tolerance, validating that AI-guided Raman-activated sorting can link single-cell phenotype to genotype [111].

The MaGIC-OT platform uses deep reinforcement learning (DRL) rather than supervised learning [104]. The platform was trained in a high-fidelity simulation environment that replicates optical trapping in microfluidic channels. A deep Q-network agent learned to navigate a trapped cell to an isolated analysis chamber using only local visual input, without requiring a global map of the environment. Cooperative training—alternating control between a human operator and the agent—improved isolation success rates from approximately 24% to 45% compared to supervised learning alone [104]. The trained agent was then deployed on a physical optical trapping system, where it successfully isolated cancer cells spiked into blood samples. The isolation path length averaged 889 ± 59 μm, with a transport speed of 15.0 ± 1.7 μm/s, corresponding to approximately 51 cells per hour [104]. Critically, the DRL agent did not require whole-field rescanning to update its environmental map; it reacted to local obstacles in real-time, making it tolerant to Brownian motion and fluidic drift that would invalidate a precomputed path.

To the best of our knowledge, no published system to date has fully integrated all three layers into a single POT-SERS platform. Table 4 summarizes the state of the art. The AI-RACS system integrates sensing and processing but requires manual target confirmation [111]. The MaGIC-OT platform achieves autonomous navigation but does not incorporate SERS feedback [104]. TrapNet and the DNH-Raman tweezer demonstrate high-accuracy classification and spectral acquisition, respectively, yet neither system provides active control over the target [77]. The critical missing component is the real-time coupling of SERS spectral feedback to hardware actuation. Achieving this integration—where an ML model processes a SERS spectrum and automatically adjusts trapping parameters or initiates sorting without human intervention—remains the central challenge for the field. Once realized, closed-loop POT-SERS will enable adaptive experimentation: the system will continuously evaluate whether a trapped particle is chemically informative and physically stable, then adjust acquisition parameters or release the particle accordingly, thereby improving throughput, eliminating subjective bias, and preserving fragile samples from excessive photodamage.

## 6. Challenges and Future Outlook

Despite the progress summarized in Table 4, the integration of AI into POT and SERS faces several challenges. Many of these limitations arise from the combined complexity of nanoscale optical trapping, plasmon-enhanced spectroscopy, and data-driven analysis.

One of the most significant challenges is the limited availability of large, high-quality labeled datasets. DL models generally require thousands of spectra per class to achieve reliable performance [84]. However, in single-entity POT-SERS experiments, collecting such datasets is inherently slow and expensive because each trapping event may require seconds to minutes, followed by careful spectral validation. For rare targets such as circulating tumor cells, extracellular vesicles, or individual viruses, acquiring sufficiently large numbers of positive samples can become impractical. In addition, the quality of labels is often uncertain because particle identity may rely on fluorescence imaging, electron microscopy, or sequencing, each of which introduces potential sources of error. Incorrect or noisy labels can propagate through the training process and reduce model robustness. To address these limitations, future research may increasingly rely on few-shot learning, transfer learning, and self-supervised learning approaches that reduce dependence on large labeled datasets [113]. In particular, pretraining models on large publicly available SERS datasets and subsequently fine-tuning them for task-specific applications represents a promising direction.

Another important limitation is the lack of interpretability of DL models. Many neural networks function as “black boxes,” producing classifications without clearly explaining which spectral features influenced the prediction [114]. In POT-SERS applications, this limitation raises concerns regarding model reliability, reproducibility, and scientific validity. A model may learn correlations associated with experimental artifacts, such as substrate variability, laser alignment, or background fluorescence, rather than genuine molecular signatures. Furthermore, limited interpretability reduces confidence among end users, particularly in biomedical and clinical settings where explainable decisions are essential. Explainable artificial intelligence (XAI) methods such as gradient-weighted class activation mapping (Grad-CAM) and SHAP (SHapley Additive exPlanations) offer partial solutions by identifying spectral regions that contribute most strongly to model predictions [115]. Nevertheless, these methods remain post hoc approximations and do not guarantee that the learned representations are physically meaningful. Future work may focus on physics-informed AI models that incorporate prior spectroscopic knowledge, including Raman peak positions, Lorentzian line shapes, and non-negativity constraints, directly into the network architecture [116]. Hybrid approaches combining DL with classical spectral fitting methods may provide an effective balance between predictive accuracy and interpretability.

Generalization across experimental platforms represents another major barrier to real-world deployment. Models trained on spectra acquired using one substrate or Raman instrument often exhibit degraded performance when applied to data collected using different experimental conditions. These discrepancies arise from variations in plasmonic enhancement factors, hotspot distributions, substrate morphology, detector sensitivity, and spectral calibration. Consequently, models may fail when transferred across laboratories or measurement platforms, limiting reproducibility and clinical translation. Domain adaptation strategies and self-supervised learning methods offer promising routes for improving cross-platform robustness by learning instrument-invariant spectral representations [117]. In parallel, the development of standardized preprocessing workflows, calibration procedures, and open-access benchmark datasets will be essential for improving reproducibility within the POT-SERS community.

Beyond data processing, a key feature of future autonomous systems is real-time closed-loop feedback, where the SERS spectrum acquired from the trapped particle is continuously processed by AI to adjust trapping parameters—optimizing stability and signal while minimizing photothermal damage, as illustrated in Figure 1. Closed-loop systems must rapidly process spectra, evaluate trapping stability, and dynamically adjust experimental parameters under fluctuating nanoscale conditions. Although trapping events typically occur over seconds to minutes, low-latency analysis remains critical for adaptive feedback control. Large transformer-based architectures and computationally intensive DL models may therefore be difficult to implement directly in portable or integrated POT-SERS systems. Future autonomous platforms will likely depend on lightweight neural networks, edge computing, and neuromorphic hardware capable of performing efficient real-time computation with reduced power consumption [118]. Such advances could facilitate the development of compact and field-deployable intelligent nanophotonic systems.

Compared with established bioanalytical technologies such as flow cytometry, imaging flow cytometry, PCR, digital PCR, and next-generation sequencing (NGS), POT and POT-SERS currently exhibit lower throughput, higher experimental complexity, and limited clinical translation. Table 5 summarizes the principal characteristics, strengths, and limitations of these approaches in comparison with conventional platforms. As a result, established methods remain the standard for routine, high-throughput clinical diagnostics. On the other hand, POT-based techniques should be viewed as complementary tools that provide access to fundamentally different measurement modalities, enabling label-free manipulation and spectroscopic interrogation of individual nanoscale entities in their native environment. To address current limitations in scalability and usability, recent developments increasingly point toward the integration of AI. In particular, AI-assisted workflows may enable automated particle recognition, adaptive trapping control, real-time spectral deconvolution, and closed-loop optimization of experimental parameters, while also facilitating inverse design of plasmonic substrates. These advances are expected to improve robustness and reduce operator dependency, thereby narrowing—but not eliminating—the gap with established high-throughput technologies. Importantly, rather than replacing conventional bioanalytical methods, AI-enabled POT-SERS platforms are expected to complement them by providing single-entity chemical and biophysical information that is inaccessible to ensemble-averaged approaches, with potential impact in precision diagnostics, mechanobiology, virology, and single-molecule biophysics.

Despite these advantages, the application of POT-SERS to clinically relevant and complex biological samples remains challenging. In biofluids such as human plasma, abundant proteins—most notably albumin—can non-specifically adsorb onto plasmonic nanostructures, leading to partial blockage of electromagnetic hotspots and a consequent reduction in signal-to-background ratio. In addition, such biofouling can alter local trapping conditions, thereby affecting measurement stability and reproducibility during extended acquisition times. These issues highlight the need for dedicated surface engineering strategies, including antifouling coatings and selective functionalization, as well as optimized microfluidic sample handling to reduce nonspecific adsorption. In parallel, AI-based denoising and spectral unmixing methods may further enhance robustness under high-background conditions.

Beyond analytical performance, clinical translation is further constrained by practical implementation barriers that must be explicitly addressed for real-world deployment. In terms of cost-per-test and workflow integration, established diagnostic assays such as ELISA remain highly standardized, inexpensive, and fully embedded in routine clinical laboratory pipelines, whereas POT-SERS currently requires advanced optical instrumentation, precise alignment, and trained operators, leading to higher operational complexity and increased cost per measurement at the present stage. In addition, although current POT-SERS implementations are primarily limited to in vitro single-entity studies, future extension toward in vivo or minimally invasive applications will require systematic biocompatibility validation. This includes assessment of the long-term stability of plasmonic nanostructures under physiological conditions, evaluation of potential cytotoxic effects, and characterization of immune responses associated with repeated or prolonged exposure to nanomaterials. Finally, successful clinical translation will depend on seamless integration with existing diagnostic infrastructures rather than replacement of established methods. This will require the development of automated microfluidic sample handling systems, standardized and interoperable data formats, and AI-assisted interpretation pipelines to enable robust operation within routine laboratory workflows. Taken together, these considerations define a clear translational pathway in which POT-SERS evolves as a complementary diagnostic modality rather than a competing platform.

**Figure 7:**
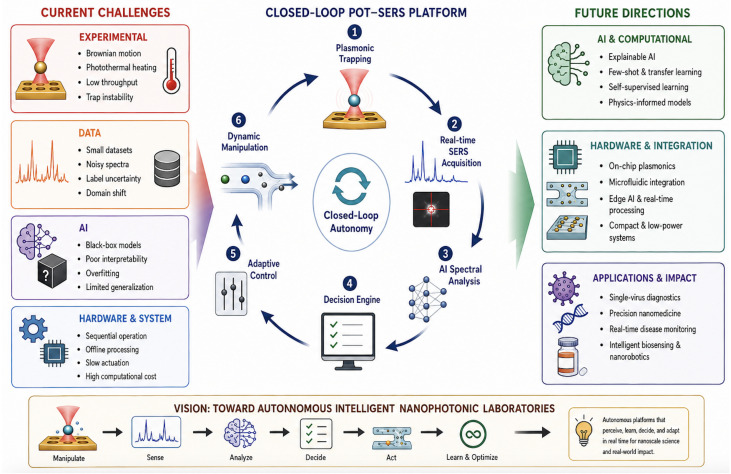
Schematic illustration of the major challenges and future research directions for intelligent POT-SERS platforms. Current limitations include low-throughput trapping, photothermal effects, noisy and heterogeneous spectral datasets, poor model generalization, and limited interpretability of deep learning algorithms. Future autonomous POT-SERS systems are expected to integrate real-time spectral acquisition, machine learning-based analysis, adaptive feedback control, and automated actuation within closed-loop nanophotonic platforms. Emerging approaches including explainable AI, self-supervised learning, inverse nanostructure design, microfluidic integration, and edge computing may enable fully autonomous nanophotonic laboratories for applications in biosensing, virology, precision diagnostics, and single-molecule biophysics. Figure created with assistance from ChatGPT (OpenAI-GPT 5.5).

The challenges outlined above remain substantial, but continued advances in nanophotonics, microfluidics, and AI are steadily driving POT-SERS platforms toward greater autonomy. Figure 7 summarizes the major challenges, emerging solutions, and future directions toward autonomous intelligent nanophotonics platforms. In the near term, semi-autonomous systems are expected to dominate, where AI supports particle identification and spectral analysis while human operators validate key decisions. Progress will depend on large open-access datasets and interpretable models that link predictions to physically meaningful molecular features. A likely next step is the adoption of conditional autonomy, whereby systems manage routine low-risk operations autonomously and seek human input when faced with uncertainty or high-stakes situations. This will require robust uncertainty quantification, domain adaptation, and reliable multi-modal integration across trapping, spectroscopy, and microfluidics, enabling higher throughput with minimal human supervision. Ultimately, fully autonomous POT-SERS platforms may emerge by integrating POT, SERS, microfluidics, and AI into closed-loop systems capable of particle trapping, spectral acquisition, real-time classification, sorting, and self-calibration. Despite remaining engineering challenges, rapid progress in AI-enabled nanophotonics suggests that autonomous nanoscale laboratories are a realistic long-term goal.

## Figures and Tables

**Table 1:** Comparison of POT architectures.

Architecture	Trapping Performance	Key Advantage	Limitation	Thermal Effects	Throughput	Application Domain
Ag@PDMS nanocavity array [52]	14 nm particles; multiple trapping sites	Low power; scalable array	Fabrication complexity	Low heating (PDMS thermal insulation)	High (multi-site parallel)	High-throughput nano-particle trapping
Bowtie-nanohole [53]	4.8 $k_{B}$T potential depth	Enantioselective chiral sorting	Chiral sorting; low throughput	Moderate (hotspot concentration)	Low (single-site)	Chiral nano-particle separation
Triple-coaxial nano-aperture [54]	20× force enhancement; 30 nm PS	Enhanced transmission (0.25 at 795 nm); resonant coupling	Complex fabrication; 3-layer structure	Moderate to high (metal absorption)	Low (single-site)	Single-particle trapping
DNH/${\mathrm{Si}}_{3}$$N_{4}$ waveguide [55]	10× force enhancement; sub-2 nm confinement	On-chip integration; no free-space optics	Fabrication complexity	Moderate (waveguide dissipation)	Low	Lab-on-a-chip integration
Fano-resonant metamaterial [56]	0.61 mW/μ$m^{2}$ trap stiffness; 20 nm Au particles SIBA-assisted trapping; high stiffness	Metamaterial fabrication complexity	Moderate (Fano resonance heating)	Low	Single-nano-particle trapping	
Hybrid dielectric–plasmonic nanobowtie [64]	>10 $k_{B}$T; 100 nm virus particles	Reduced heating; high stability	Hybrid fabrication complexity	Low (dielectric reduces heating)	Low	Virus trapping; biological applications
Gold nanoarray (graded gaps) [49]	97% capture efficiency	Label-free continuous sorting	Sorting only; limited trapping versatility	Moderate (metal absorption)	Moderate	Particle sorting
Plasmonic multi-slot array [60]	20 nm particles; 70 nm step displacement	Dynamic transport; precise inter-particle control	Multi-wavelength excitation needed	Moderate to high (multi-slot heating)	Low	Dynamic particle manipulation

**Table 2:** Comparison of integrated POT-SERS platforms.

Platform	SERS Sensitivity	Laser Power	Thermal Management	Throughput	Key Limitation
Thermoplasmonic tweezer [74]	Single-molecule (bianalyte statistics)	0.1 mW/μ$m^{2}$	Thermophoretic cooling	Low	Complex assembly dynamics
Bull’s eye nanostructure [75]	Trap stiffness correlation with SERS	8 mW	Moderate	Low	Requires calibration; 5 μm beads
Dynamic plasmonic nanocavity [76]	$10^{9}$ enhancement; single-molecule	8 mW (1064 nm trap) + 2.7 mW (532 nm SERS)	Active hotspot control reduces denaturation	Very low	Complex dual-trap setup
DNH Raman tweezer [17]	400 counts/s; single nano-particle	14 mW	Moderate	Low	Commercial Raman system required
Interferometric Electrohydrodynamic Tweezers [72]	Single sEVs characterization	—AC field reduces heating	High (12,321 sites)	Single-molecule sensitive	

**Table 3:** Comparison of AI methodologies for SERS and POT data analysis.

Method	Category	Key Advantage	Limitation	Accuracy	Primary Application
PCA [20,85,86]	Unsupervised ML	Dimensionality reduction; interpretable loading vectors	Linear; sensitive to outliers	61.7% variance captured	sEVs classification; virus detection
1D CNN [92]	Supervised DL	Automatic feature extraction; no manual preprocessing	Requires large labeled datasets	98.27%	Cancer detection from serum SERS
ResNet [93]	Supervised DL	Deep feature learning; gradient preservation	Black-box nature; computationally intensive	>99%	Small-molecule binding site screening
Variational autoencoder [91]	Unsupervised DL	Generative; identifies spectral features	Complex architecture; requires tuning	99.3%	Antibiotic susceptibility testing
TrapNet (CNN+KAN) [77]	Supervised DL	Robust to domain shifts; nonlinear feature extraction	Classification only; no active control	99%	sEVs classification from trapping signals
Deep reinforcement learning [104]	Reinforcement learning	Adaptive control; no global map required	Requires simulation training; sample inefficient	—	Autonomous navigation; cell isolation
Bidirectional neural network [101]	Supervised DL	Inverse design capability; 20× faster than conventional	Focused on structured light; indirect POT relevance	95.4%	Plasmonic vortex analysis; optical design
KNN [88]	Supervised ML	Simple; interpretable; computationally efficient	Limited by feature engineering; lower accuracy	85.7% sensitivity	Clinical virus screening

Abbreviations: CNN: convolutional neural network; KAN: Kolmogorov–Arnold network; KNN: k-nearest neighbors; ResNet: residual neural network.

**Table 4:** Comparison of autonomous and semi-autonomous platforms for optical trapping and SERS.

Platform	Trapping Method	SERS Integration	ML Method	Capability	Closed-Loop
AI-RACS [111]	OT	Yes (Raman-activated sorting)	CNN (object detection)	Raman sorting	Semi (manual target confirmation)
MaGIC-OT [104]	OT	No (visual only)	Deep reinforcement learning	Autonomous control	Yes (autonomous navigation)
TrapNet [77]	DNH OT	No (transmission only)	CNN + KAN	Classification	No (classification only)
S-RACE [112]	Laser-induced forward transfer	Yes (stimulated Raman)	None (threshold-based)	High speed sorting	Semi (real-time imaging-sorting)
DNH-Raman tweezer [17]	DNH OT	Yes (SERS)	None	Single-entity SERS	No

Abbreviations: S-RACE, stimulated Raman-activated cell ejection.

**Table 5:** Qualitative comparison of POT/POT-SERS with representative bioanalytical technologies.

Technology	Throughput	Automation	Primary Information	Advantages	Limitations
Flow cytometry [119]	Very high	High	Cell phenotyping	High throughput clinical standard	Limited molecular detail; labeling often required
Imaging cytometry [120]	High	High	Morphology fluorescence	High content single cell imaging	Required labeling; limited chemical specificity
PCR/dPCR [121]	High	High	Nucleic acid quantification	Very high sensitivity; clinical standard	Target specific; no structural info
NGS [122]	Moderate high	High	Genomic profiling	Comprehensive molecular readout	Costly; destructive; complex analysis
POT-SERS [1,11]	Low	Emerging	Single-entity trapping and Raman fingerprint	Label-free simultaneous manipulation spectroscopy	Low throughput; photothermal effects; complex setup

## Data Availability

No new data were created or analyzed in this study. Data sharing is not applicable to this article.
